# Staining of a Conventional and a Nanofilled Composite Resin Exposed *in vitro* to Liquid Ingested by Children

**DOI:** 10.5005/jp-journals-10005-1074i

**Published:** 2010-09-15

**Authors:** Amit Khatri, B Nandlal

**Affiliations:** 1 Lecturer, Department of Pedodontics, University College of Medical Sciences, Delhi University, New Delhi, India; 2 Head, Department of Pedodontics and Preventive Dentistry, JSS Dental College and Hospital, Mysore, Karnataka, India

**Keywords:** Resin composite, Color, Staining.

## Abstract

**Aims and objective :** To evaluate and compare the effect of coffee, chocolate drink and food dye on the color stability of conventional composite resin and nanocomposite resin.

**Design :** The study sample consists of microhybrid composite and other nanocomposite of shade B1. 40 disks of each material with a diameter of 18 mm and thickness of 1 mm were prepared before discoloration procedure specimens were measured for L* a* b* values and then 10 specimens of each subgroup were immersed in each of three staining solution. The 10 remaining specimens of each main group were immersed in control solution.

Discoloration values were analyzed at baseline, 1 week and 4 weeks following immersion in staining solution. The color and color difference of each specimen were measured by spectrophotometer.

L* a * b* values of each specimens were measured 3 times by placing each specimen on the measuring head. Resistance to staining effect is expressed in ΔE unit and was calculated from the mean ΔL* a* and Δb* values for each specimens with the following formula.

**Equation :** ΔE* = [(ΔL*)^2^ + (Δa*) + (Δb*)^2^]½.

**Result :** Result of study showed that nanocomposite is more stain resistant and color change ΔE*. Conventional composite demonstrates unacceptable color change ΔE* more especially in specimens immersed in coffee.

**Conclusion :** The result of this study indicated tested conventional composite resin was more susceptible to change in color in various media over an extended period of time as compared to nanocomposite.

## INTRODUCTION

Resin composite materials have been used for many years in pediatric dentistry with great success and high patient acceptance. The color of esthetic restorative is one of the most important factors. Dental esthetic is desired by both children and parents, and the advent of tooth colored restoratives material has been indispensable for this purpose. A common problem encountered with these materials, after month and year of use and exposure to a variety of different food and liquid ingested by children.^1^ The physical properties of composite resins are dependent on the nature of resin matrix, filler particle and resin-filler interface. Physical properties can also be influenced by the chemical environment present in the mouth. The change in oral environment that causes the staining can occur either intrinsically or extrinsically. Intrinsically, color can change due to physiochemically alterations of resin matrix itself within resin matrix also. Color can also change extrinsically due to absorption of stains on the outer surface.^1^

Many new materials have been developed, and the ability to prevent extrinsic and intrinsic stains of restorations has become an important challenge.

The most recent innovation in composite resin technology is the revolutionary application of nanocomposite theories. Contemporary nanocomposite material delivery increases esthetic, strength and durability, which are scientific principles for increased longevity.

Development of nanocomposite resin using advanced methacrylate resin has esthetic properties required for anterior restoration and mechanical properties required for posterior restoration. Nanofillers are different from traditional fillers. Milling procedure cannot reduce the filler particle size below 100 nm (1 nm = 1/1000 um). Synthetic chemical processes are used to produce building block on molecular scale. The nanomeric particles are monodisperse non- aggregated and non-agglomerated silica nanoparticle.^2^ Tooth color composite resin and resin-based veneering material have been unable to retain the color they posses at the time of insertion, and lack color stability.

Spectrophotometric has made it possible to study the numerous parameter related to the color stability of composite.^3^ It is of interest to investigate the color stability of some of conventional composite and recently introduced nanocomposite as veneer or restorative material. This study was conducted to check the color stability of a conventional composite and nanocomposite in various staining solutions.

## MATERIALS AND METHODS

Two different types of restorative resin conventional composite [microhybrid] (TPH-Spectrum) (Dentsply B1) shade and nanocomposite (Ceram-X B1) shade were evaluated after immersion in three staining solutions and in artificial saliva as control.

Forty disk of each material with a diameter of 18 mm and thickness of 1 mm were prepared. The disks were made by placing the resin in Teflon mold 1 mm thick and and 18 mm in diameter. The resin was carefully packed ensuring that mold was completely filled. Extramaterial was expelled by pressing down with a glass slide.^4^

The specimen’ lower side was light cured with a halogen curing lite (model no XL 3000, 3M, St. paul Minn) for 20 seconds. The specimens were turned over and the opposite side was also light cured for 20 seconds.^5^

The LED curing light was calibrated before and after each curing to ensure that all samples were cured with approximately the same intensity of light (500 mW/cm^2^). The light was held at the same distance for each episode of curing to ensure that all samples are cured under uniform conditions.^6^

The specimen were then incubated in 100% humidity at 37° for 24 hours before baseline measurement was taken. The incubator did not allow exposure to ambient light. Following the preparation of the composite resin specimens, they were polished with a high-speed carbide no 7901 bur and Sof-lex (3M ESPE) composite resin polishing disk. The carbide finishing bur was used to replicate the initial finishing in clinical situations. They were then polished with black coarse so flex disk for 90 seconds at speed of 5,000 rpm followed by medium blue fine disk for 30 seconds at 5,000 rpm.^6,7^

Staining solution was also prepared. To prepare coffee staining solution (Nescafe classic, Nestle, Nanjangud, Mysore) 15 gm of coffee was poured into 500 ml of hot water and filtered after 10 minutes, prior to being poured into container.^8^

Similarly, 15 gm of food dye (yellow/orange) was poured into 500 ml of hot water and poured into container other solutions used were synthetic saliva (Wet mouth. ICPA health products, Ankleshwar) and chocolate drink (Fun Foods Pvt. Ltd, Alwar).

A total of 80 samples were evaluated. Before discoloration procedure specimens were measured for L* a* b* values and then 10 specimens of each subgroup were immersed in each of three staining solutions. 10 remaining specimens of each main group were immersed in control solution.

After polymerization, a length of dental floss was attached to the edge of resin disk specimen with cyanoacrylate to permit suspension in solution. Each specimen was suspended in 12 ml of solution so that it did not contact the container or other disk. Each disk was identified by a letter and number written on plastic bottles.^9^

The staining solutions and the control solution containing the specimens were placed in a thermostatically-controlled incubator at 37° ± 1 to simulate conditions in oral cavity.

The solutions were stirred frequently to prevent sedimentation of stained solutions. The solutions were changed every week to prevent fungal contamination.

The test specimens were placed in the container and removed for testing with sterile forceps. After removal from the solutions, specimens were cleaned ultrasonically with distil water for 1 minute and then blotted dry with tissue paper.^10^ Discoloration values were analyzed at baseline, 1week and 4 weeks following immersion in staining solution. The color and color difference of each specimen were measured by spectrophotometer. (Gretag Macbeth). The testing apparatus had a measuring head diameter of 15 mm the measuring characteristics of spectrophotometer were standard illuminant D65, illuminating view geometry d/10. The samples were kept against a white background during all measurements. This ensures that the spectrophotometer was used with a consistent background for each sample and prevent variability due absorption or any other confounding color effects. Before each measurement spectrophotometer was calibrated according to manufacture recommendation. The calibration was done against white standard (supplied with instrument) with known color dimensions.

Values were recorded in commission internationale del’ eclairage (CIE). CIE L*a*b* color system. The CIE L*a*b* system is an approximately uniform color space with coordinates for lightness, namely white/black (L), Red/green (a) and yellow/blue (b). L*a*b* values of each specimens were measured 3 times by placing each specimen on the measuring head. The values of ΔL* Δa*and Δb* after three measurements were automatically calculated by spectrophotometer and recorded. Thus ΔE* was more meaningfull then individual L*a*b* values. Resistance to staining effect is expressed in ΔE* unit and calculated from the mean ΔL* Δa* and Δb* values for each specimens with the following formula;

Equation: ΔE* = [(ΔL*)^2^ + (Δa*) + (Δb*)^2^]½

## RESULTS

All results for both conventional composite and nanocomposite were submitted to parametric analysis. Difference in L*a* b* values before and after colorations were tested with repeated measure ANOVA and paired ‘t’ test.

The staining of samples was characterized by a clear drop in L*. It was found that L*a* value significantly decreased in samples immersed in coffee in both groups. Decrease in value of L* was more observed in conventional composite group compared to nanocomposite group.

It was found that b* value significantly increases in samples immersed in coffee in both the groups. Increase in the value of b* was more observed in conventional composite group.

The color change ΔE* observed between the composite and nanocomposite and the staining media during the study was subjected to analysis of variance and paired ‘t’ test for ΔE* value comparison at different time intervals.

Between the groups as well as staining solutions, a highly significant difference was found (P < 0.001).

**Fig. 1:**
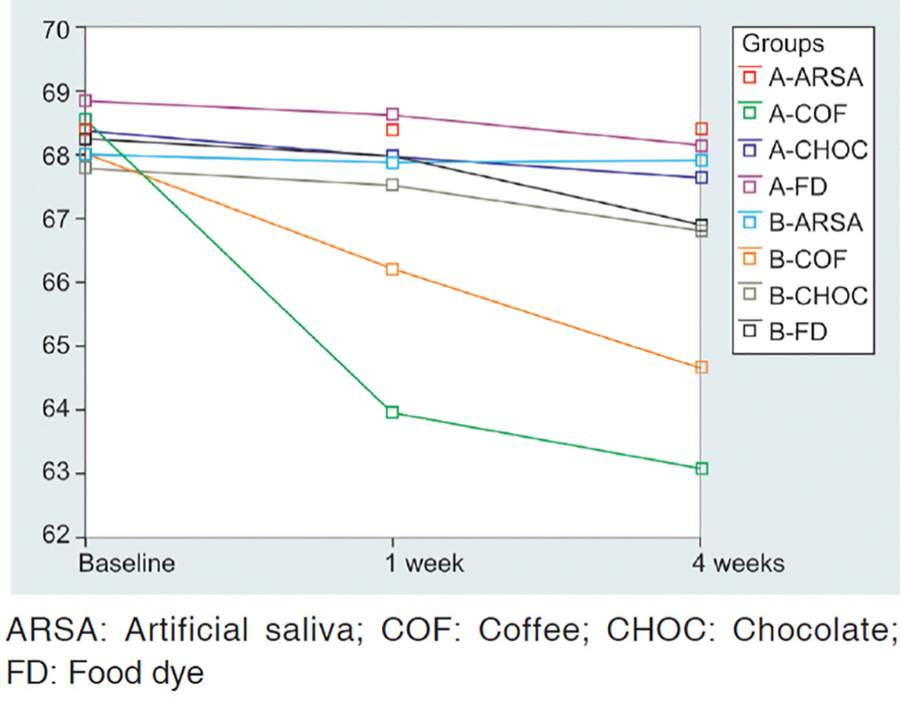
Mean L* values measurement following sample staining of both conventional composite group and nanocomposite group over a period of 4 weeks

**Fig. 2:**
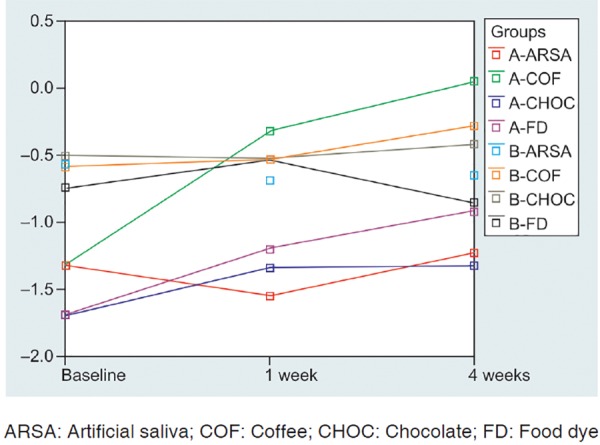
Mean a* values measurement following sample staining of both conventional composite group and nanocomposite group over a period of 4 weeks

**Fig. 3:**
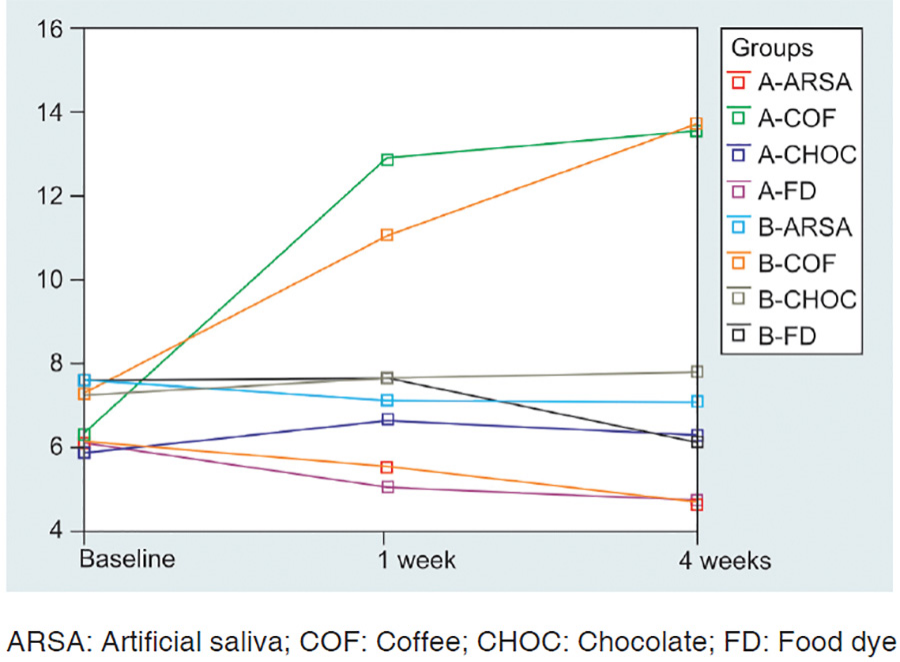
Mean b* values measurement following sample staining of both conventional composite group and nanocomposite group over a period of 4 weeks

Maximum color change was observed after the first week of immersion. It gradually increased from 1 to 4 weeks. The results of the study are summarized in Tables 1 to 4 and Figures 1 to 3.

**Table 1:** **Table 1:** Descriptive statistics for color parameters (L*) in conventional composite group and nanocomposite group and solutions before (baseline) and after immersion in four staining solutions

		*Baseline*		*Artificial saliva*				*Coffee*				*Chocolate*				*Food dye*			
				*1 week*		*4 weeks*		*1 week*		*4 weeks*		*1 week*		*4 weeks*		*1 week*		*4 weeks*	
Conventional		68.54		68.37		68.41		63.97		63.8		67.98		67.63		68.63		68.12	
composite (L*)		(0.52)		(6.27)		(0.67)		(0.66)		(0.98)		(0.37)		(0.82)		(0.76)		(0.99)	
Nanocomposite		68.01		67.87		67.91		66.22		64.66		67.52		66.90		67.96		66.90	
(L*)		(0.54)		(0.62)		(0.58)		(0.58)		(0.88)		(0.70)		(0.47)		(0.53)		(0.48)	

The quality L* correlates to lightness, the light value coordinates L* ranges from 0 = black to 100 = white similar to value in the Munsell system

## DISCUSSION

Tooth color polymeric materials have been unable to retain the color they possess at the time of insertion. Color stability can be affected by physical nature of food, e.g. solid or liquid, cold or hot. Other causes may include microcracks on the materials, crazing, porosity free radical having solution affinity for certain pigment. Color pigment found in tea, coffee and food dyes, including turmeric causes color change.^11,12^

The nature of resin also accounts for some difference in staining potential.^13^ Resin material incorporate urethane dimethacrylate seems to be more stain resistant than resin matrix using dimethacrylate as a matrix.

**Table 2:** **Table 2:** Descriptive statistics for color parameters (a*) in conventional composite group and nanocomposite group and solutions before (baseline) and after immersion in four staining solutions

		*Baseline*		*Artificial saliva*				*Coffee*				*Chocolate*				*Food dye*			
				*1 week*		*4 weeks*		*1 week*		*4 weeks*		*1 week*		*4 weeks*		*1 week*		*4 weeks*	
Conventional		– 1.50		– 1.54		– 1.22		– 0.31		– 0.30		– 1.34		– 1.32		– 1.2		– 0.91	
composite (a*)		(0.74)		(0.14)		(0.89)		(0.22)		(0.46)		(0.11)		(0.13)		(0.16)		(0.21)	
Nanocomposite		– 0.59		– 0.68		– 0.65		– 0.54		– 0.28		– 0.51		– 0.41		0.53		0.84	
(a*)		(0.54)		(0.34)		(0.40)		(0.33)		(0.31)		(0.31)		(0.47)		(0.32)		(0.79)	

The a* coordinate describe the chromatic component. The a* defines the color green (negative values) and color red (positive values)

**Table 3:** **Table 3:** Descriptive statistics for color parameters (b*) in conventional composite group and nanocomposite group and solutions before (baseline) and after immersion in four staining solutions

		*Baseline*		*Artificial saliva*				*Coffee*				*Chocolate*				*Food dye*			
				*1 week*		*4 weeks*		*1 week*		*4 weeks*		*1 week*		*4 weeks*		*1 week*		*4 weeks*	
Conventional		6.12		5.54		4.64		12.84		13.54		6.65		6.31		5.12		4.7	
composite(b)*		(0.58)		(1.00)		(0.76)		(1.01)		(0.65)		(0.33)		(0.72)		(0.87)		(0.75)	
Nanocomposite		7.44		7.14		(7.09)		11.03		13.67		7.60		7.7		7.67		6.22	
(b)*		(0.65)		(0.89)		(0.91)		(1.4)		(1.00)		(0.43)		(0.53)		(0.72)		(1.05)	

The b* coordinate describe the chromatic component. b* defines the amount of yellow (positive value) and blue (negative values)

**Table 4:** **Table 4:** Descriptive analysis of ΔE* color difference between immersed samples (mean) and baseline over a period of 4 weeks

		*Conventional composite*				*Nanocomposite*			
*Staining solution*		*1 week*		*4 weeks*		*1 week*		*4 weeks*	
Artificial saliva		0.87 (0.38)		1.79 (0.41)		0.55 (0.16)		0.75 (0.17)	
Coffee		8.2 (0.87)		9.36 (1.37)		4.18 (0.95)		7.2 (1.15)	
Chocolate		0.99 (0.32)		1.47 (0.53)		0.54 (0.18)		1.20 (0.36)	
Food dye		1.27 (0.51)		2.73 (1.4)		4.1 (0.35)		2.05 (0.07)	

The structure of composite and characteristic of the particle have a direct impact on the surface smoothness and susceptibility to extrinsic staining.^14^

The high staining susceptibility of hybrid composite resin could be attributed primarily to its high resin contents and the related water contents. It has been demonstrated that diffusion coefficient of water resorption in hybrid composite is within the range of reported for conventional composite resin.^15^

The sizes of filler particles also affect the gloss retentions and high gloss surface generally considered less susceptible to staining.^16^ Microhybrid composites have an average filler particle size of approximately of 1 urn or less. Therefore, these materials have high filler loading to yield high mechanical properties and low polymerization shrinkage while maintaining smooth surface after polishing. The average cluster sizes of the nanofilled composite resin are fundamentally different conventional composite filler. The material was created using two fillers: nanomers (i.e. individual filler particle, roughly spherical in shape) and nanocluster (i.e. loosely agglomerate collection of nanoparticle) that contribute to polish retention and control of transparency because the nanomer and nanocluster fillers are combined directly with a reduced shrinkage resin. These are types of composite in which the primary filler range is in nm range while secondary filler clusters are in um range. This particle cannot be further subdivided under normal abrasive forces in the mouth in contrast to nanosized particle in nanocomposite wear by breaking of individual primary particle rather than plucking out larger secondary particle from resin, thus the resulting wear surface has better gloss retention.^17^ Since the nanotechnology allows a higher level of control, a better color stability of nanocomposite was expected. In the present study, color stability of conventional composite resin and nanocomposite resin were evaluated. Study was carried out to determine the color change, i.e. ΔE* between groups after staining, to compare the amount of change occurring between the groups and to determine the staining potential of coffee, chocolate and food dyes.

There were many reasons for choosing these solutions as test agents because these are commonly ingested by children. Firstly, although there have been several studies evaluating the ability of composite to resist staining from food and beverages, most of these have involved the assessment of ‘adult’ type of beverage (red wine, tea). Artificial saliva as control and chocolate drink contains coffee as ingredient and so these solutions were chosen as staining agent.^18^

Secondly, the choice of staining solution in this study attempted to represent diverse area of color spectrum. Artificial saliva is colorless, coffee is light brown and chocolate is dark brown and food drink is yellow/orange. There are differences in consistency as well. Coffee solution and food dye is thin, watery solution whereas chocolate is thick and syrupy.

All samples were immersed in staining solution of coffee, chocolate and food dye. In the present study, 1 week was selected as test period because one previous study found that staining effect after 1 week of coffee immersion differed significantly from all succeeding weeks and at that point staining reached a pleatue. Similar findings were found in the present study and maximum change in color was observed in first week. In earlier related studies, it has been shown that most of the water sorption takes place during the first week. This certainly accounts for the mainly insignificant increase in samples color change observed between 1st and 4th week.^16^ Change in color by coffee was due to adsorption, and also due to absorption of colorants by two of the materials investigated. This absorption and penetration of colorants into the organic phase of the veneering materials were probably due to compatibility of polymer phase with the yellow colorants of coffee.^13^

According to p-value, highly significant differences were found among values for solutions, same occurred with materials (P < 0.001). Paired t-test revealed highly significant difference in L* a* b* values between baseline to 4 weeks in both the composite and nanocomposite groups in all staining solutions.

In the previous studies, it was concluded that ΔE* values greater than or equal to 3.3 were considered visually perceptible. Less than this is clinically insignificant.^41,93^ In our study, both conventional composite and nanocomposite exhibited a ΔE* value over 3.3 in coffee staining solution, conventional composite and nanocomposite did not exhibit a ΔE* value over 3.3 in other solutions like artificial saliva, chocolate and food dye after the 4 weeks of testing.

In the present study, maximum discoloration was observed during the first week followed by 4 weeks. Although color change was observed maximum in 1 week it gradually increased from 1 week to 4 weeks.

After evaluating the color parameters (L*a* b*) and total discoloration (ΔE*) of both conventional composite and nanocomposite over a period of 4 weeks, it was revealed that nanocomposite was more stain resistant and maximum discoloration (ΔE*). Conventional composite demonstrates unacceptable color change ΔE* more than eight especially in specimens immersed in coffee as compared to nanocomposite. Coffee had the highest staining potential in both conventional composite and nanocomposite group.

The result of the present study indicated conventional composite resin is more susceptible to discoloration in various media over an extended period of time compared to nanocomposite.

Result of present investigation suggests that similar may occur clinically and indicates the importance of patient habit in the longevity of restorative materials. However, further studies are needed to determine whether the color of tested resin composite would be stable in long-term clinical situations or not. Furthermore, the staining potential of both conventional composite and nanocomposite with other staining solutions should be tested.

## CONCLUSION

1. Conventional composite resin is more susceptible to discoloration in various media over an extended period of time as compared to nanocomposite.
2. It was concluded that maximum discoloration observed during the first week followed by 4 weeks. Color change gradually increases from 1 to 4 weeks.
